# Exploring Patient and Caregiver Work and Continuity of Care During Hospital‐to‐Home Transitions of Care in the Canadian Context

**DOI:** 10.1111/hex.70727

**Published:** 2026-06-18

**Authors:** Hardeep Singh, Michelle L. A. Nelson, Terence Tang, Kerry Kuluski, Anie Udofia, Hedieh Molla Ghanbari, Ingryd Cunha Ventura Felipe, Jason Nie, Kednapa Thavorn, Robert J. Reid, Walter Wodchis, Carolyn Steele Gray

**Affiliations:** ^1^ Department of Occupational Science & Occupational Therapy, Temerty Faculty of Medicine University of Toronto Toronto Ontario Canada; ^2^ Rehabilitation Sciences Institute, Temerty Faculty of Medicine University of Toronto Toronto Ontario Canada; ^3^ Bruyère Health Research Institute Ottawa Ontario Canada; ^4^ Institute of Health Policy, Management and Evaluation, Dalla Lana School of Public Health University of Toronto Toronto Ontario Canada; ^5^ Institute for Better Health Trillium Health Partners Mississauga Ontario Canada; ^6^ Design (MDes) OCAD University Toronto Ontario Canada; ^7^ Division of Hospital Medicine, Department of Family and Community Medicine University of Toronto Toronto Ontario Canada; ^8^ Science of Care Institute, Lunenfeld Tanenbaum Research Institute, Sinai Health Toronto Ontario Canada; ^9^ Ottawa Hospital Research Institute Ottawa Ontario Canada; ^10^ School of Epidemiology and Public Health University of Ottawa Ottawa Ontario Canada

## Abstract

**Background:**

Patients and caregivers are expected to engage in work that involves both physical and cognitive tasks to manage their care and well‐being during hospital‐to‐home transitions. Patient and caregiver work is defined as the health‐related tasks they must manage, as well as the physical and cognitive effort required to complete those tasks. When continuity of care is well‐supported, work for patients and caregivers may be reduced or better managed. However, there is a need to better understand how continuity of care affects patients' and caregivers' work to optimise their outcomes.

**Objective:**

To explore the work undertaken by patients and caregivers during hospital‐to‐home transitions in Ontario, Canada, and examine how different dimensions of continuity impact this work.

**Design:**

This qualitative interpretive description study engaged older adults (60+) who self‐reported complex care needs and had ≥ 1 hospital‐to‐home care transition, and caregivers supporting these transitions in a semi‐structured, audio‐recorded interview. A framework analysis integrating inductive and deductive coding, based on Patient Work and Continuity of Care models, was used to analyse the data.

**Results:**

Ten participants were interviewed: four caregivers, five patients and one individual who identified as both patient and caregiver. Two themes were generated: (1) physical and cognitive work performed by patients and caregivers during hospital‐to‐home transitions, and (2) how informational, management and relational continuity impact patient and caregiver work.

**Conclusion:**

While all patient and caregiver work cannot be eliminated, their tasks may be reduced or better supported through improvements in continuity. By clarifying physical and cognitive work tasks and interpreting them through the lens of continuity of care, we were able to distil work into nine challenges that support translation of these themes into actionable interventions. Interventions that strengthen information transfer, clarify care plans, and provide targeted training and resources have the potential to lessen avoidable workload and improve transition experiences for patients and caregivers with complex care needs.

**Patient or Public Contribution:**

An advisory committee of patients with complex care needs and caregivers informed data collection (e.g., reviewed interview guide) and study output (vignette video).

**Clinical Trial Registration:**

ClinicalTrials.gov NCT04287192; https://clinicaltrials.gov/ct2/show/NCT04287192.

## 1 Introduction

Hospital‐to‐home transitions can be challenging for older adults with complex care needs and their caregivers due to multiple chronic conditions along with social and/or mental health challenges that make management of their health conditions challenging [1, 2]. Moreover, these individuals may be especially vulnerable due to increased demands for care at home and in the community following hospital discharge [3]. Hospital‐to‐home transitions usually require extensive coordination among health and social care clinicians and professionals across settings [4, 5]. Older adults with complex care needs often experience medication management safety events, coordination failures, and communication gaps [4, 5, 6] combined with physical deconditioning and social isolation [4, 5, 7, 8, 9]. Many adverse events are preventable and may result from gaps in care continuity [10, 11, 12, 13].

Continuity of care is defined as ‘the extent to which services are received as part of a coordinated and uninterrupted succession of events consistent with the medical care needs of patients’ [14, 15]. Three types of continuity have been identified: informational continuity (the sharing of health‐related information across clinicians and healthcare events over time), management continuity (the coordination of services through complementary and timely treatment plans), and relational continuity (the maintenance of ongoing relationships with care team member(s) across episodes of care) [16]. However, achieving continuity of care is hindered by poor information flow and lack of communication, coordination, and collaboration between hospital and community clinicians [17]. Optimising continuity during these transitions can reduce care fragmentation and better support patients and caregivers [17].

The work performed by patients and caregivers in healthcare is often underrecognized [18, 19]. In this paper, ‘patient and caregiver work’ refers to the multiple health‐related tasks these individuals must manage during transitions, as well as the physical and cognitive effort required to complete them [20, 21]. The nature of patient and caregiver work varies depending on geographic location, specific health conditions (alone or in combination) and care setting [22] but both patients and caregivers are likely to experience significant added tasks as they navigate the complexities of moving from the hospital‐to‐home [23]. Caregivers play an essential role in care at home after discharge by supporting tasks ranging from medication management, wound care and monitoring the patient's health status [24]. Increased time demands for this additional work and effort can frequently exceed caregivers' capacity during transitions [21] While not all additional work is unreasonable or unavoidable, many individuals find it overwhelming due to being poorly prepared and not having adequate resources [22, 24]. Many caregivers have reported being unprepared to take on the new responsibilities associated with supporting a transition home from the hospital [24]. While studies have begun to examine the workload involved in managing chronic health conditions [25, 26, 27], the specific patient and caregiver work during hospital‐to‐home transitions remains underexplored [28], especially in the context of Canada's publicly funded healthcare system [29]

Improving processes for care transitions that enhance continuity is essential to making transition management easier for patients and caregivers [17, 24, 30]. This is particularly important during the transition from hospital‐to‐home, where fragmentation is most evident and the burden of work for patients and caregivers often increases [17, 24, 30]. Several studies focusing on transitions of care have emphasised the importance of addressing gaps in continuity to optimise these transitions for older adults and caregivers [31, 32, 33, 34]. There have also been calls for a better understanding of the connection between continuity of care and patient and caregiver work [19, 35]. To address this gap, this study aims to explore the work undertaken by patients and caregivers during hospital‐to‐home transitions in Ontario, Canada, and to examine how different dimensions of care continuity impact this work. Specifically, this study poses two research questions: 1) What work do patients and caregivers undertake during hospital‐to‐home care transitions? 2) How does informational, management and relational continuity impact patient and caregiver work during these transitions?

## 2 Materials and Methods

### 2.1 Design

A qualitative interpretive description study [36] was conducted using a pragmatic approach [37]. Interpretive description is well‐suited for applied health research, where the goal is to generate practical insights to inform clinical practice [36]. Pragmatic approaches are often used to generate actionable insights for health system improvement from understanding real‐world healthcare experiences [37]. Research ethics approval was obtained from the Research Ethics Boards of Sinai Health System and Trillium Health Partners. Participants provided informed consent verbally prior to data collection.

### 2.2 Participants

Older adults (aged 60 and older) who self‐reported complex care needs (defined as multiple chronic conditions along with social and/or mental health challenges that make management of their health conditions challenging) [2] and had experienced at least one hospital‐to‐home care transition (experienced at any time) in Canada, or those who supported an older adult as a caregiver during such a transition, and could provide informed consent, were eligible for this study. We defined older adults as those aged 60 years and older to align with previous literature on care transitions [1, 38]. Participants were recruited through purposive sampling, using a recruitment flyer distributed within patient advisory groups at two hospital network organisations in Southern Ontario.

### 2.3 Setting

This study was conducted in Canada. Canada has a publicly funded healthcare system that provides universal coverage for primary health care services and medically necessary hospital care. Community‐based services, such as some home care services, may also be publicly funded after hospital discharge, however, eligibility and availability can vary by province and region [29, 39]. Patients and caregivers may supplement these services with private health insurance plans [29, 39].

### 2.4 Data Collection

Participants shared their transition experiences through semi‐structured interviews conducted over Zoom using an interview guide (Appendix A). The interviews were conducted between June and July 2021 and audio‐recorded on Zoom by HS (a woman occupational therapist and post‐doctoral fellow at the time of the interviews).

### 2.5 Data Analysis

A framework analysis was conducted [40] because it is well‐suited for pragmatic and applied qualitative research [37, 41] is compatible with interpretive description [42] and can be used in studies guided by previous frameworks/theories [43, 44]. The analysis process involved transcription (stage 1) and familiarisation with the data through multiple readings (stage 2). Transcripts were first inductively coded (e.g., poor communication among patients, caregivers, and clinicians; confusion about discharge instructions) to identify nine challenges. Following inductive coding, HS developed a coding framework using aspects of Patient Work [25] (e.g., cognitive work, physical work) and the continuity of care framework (e.g., informational, management, and relational continuity) [16] to interpret the challenges through the lenses of continuity and patient and caregiver work. HS independently coded all transcripts into an Excel matrix following this coding framework. CSG (woman; Senior Scientist with expertise in qualitative health research) independently applied this framework to two of the transcripts (stage 3). HS and CSG then met to discuss their coding, including any discrepancies. Some codes were merged (e.g., emotional work was combined with cognitive work because these often overlapped and were less visible than physical work) or refined (e.g., contextual factors). They also discussed preliminary interpretations (stage 4). HS then applied the refined coding framework to all transcripts (stage 5; see Table 1) and summarised data by category within the framework for each transcript (stage 6). HS and CSG interpreted data into themes representing ‘a patterned meaning within data’ [45] by comparing the data across the matrix as well as within individual participants to identify patterns related to how patient work is influenced by gaps in continuity of care, through discussions and analytic notes (step 7). The Standards for Reporting Qualitative Research checklist was followed to guide the reporting process [46] Grammarly was used to check spelling, grammar, and punctuation, and to improve manuscript clarity.

**Table 1:** Coding framework based on patient work [20, 25] and continuity of care literature [16].

Code	Description
Patient work [20, 25]	
Cognitive work	Cognitive tasks associated with managing health, including emotional labour
Physical work	Physical tasks associated with managing health
Contextual factors	Contextual factors impacting physical or cognitive work e.g., patient's hearing, living situation
Continuity of care [16]	
Informational continuity	Information shared among care team members and from one health event to another over time
Management continuity	Service delivery is coordinated, complementary and timely with shared management plans over time
Relational continuity	Ongoing relationship with clinician(s) across episodes of care

### 2.6 Reflexivity

Given the nature of interpretive description [36] we acknowledge that the researchers' clinical and research backgrounds, along with their underlying assumptions about care transitions, influenced the data collected and analysed. For instance, one key assumption was that participant perspectives are valuable and can inform improvements to health services; this assumption was central to the study's design and the focus on patient and caregiver perspectives. The questions we asked were refined with input from an advisory committee comprising patients with complex care needs and caregivers, who provided feedback on the clarity, relevance, and readability of the interview questions. In addition, the research team provided feedback on the appropriateness of the questions in relation to the research aims, drawing on their research and clinical expertise in care transitions. Our perspectives may have influenced the findings by emphasising certain interpretations of participants' experiences (e.g., actionable aspects, assumptions about what patients consider ‘work’). These interpretations were discussed during team meetings, and alternative interpretations were considered throughout the analysis.

## 3 Results

### 3.1 Description of Participants

Ten participants took part in this study (see Table 2): four were caregivers (average age: 62 ± 17), five were patients (average age: 71 ± 7), and one was both a patient and a caregiver (age: 64). Six were women and four were men. All participants lived in urban or suburban areas of Canada and spoke English fluently. Participants identified as: white (European, North American; *n* = 7), South Asian (*n* = 2) and Southeast Asian (*n *= 1). Most participants (*n* = 9) had a college or university‐level education. Among the eight participants who responded, six had family income before tax of ≥ $80,000 CAD. One had an income under $50,000 CAD, and the other had an income of $60,000 CAD.

**Table 2:** Participant demographics.

Code: Caregiver (C) or patient (P)	Age	Sex, Gender	Employment status	Live alone?	Born in Canada?	Language spoken at home	Ethnic group	Highest education level	Chronic condition details
P1: C	70	Female, Woman	Retired	Yes	Yes	English	European‐White	College or University (undergraduate)	Heart disease, arthritis, chronic pain, displaced disc
P2: C	81	Male, Man	Retired	No	No‐India	Gujarati	South Asian	Graduate or professional	Heart disease, cholesterol, diabetes
P3: P	61	Male, Man	Employed	No	Yes	English	North American‐White	College or University (undergrad)	Gout‐occasional, coeliac, aneurysm, heart disease, cancer, chemo last fall and surgery
P4: C	56	Female, Woman	Employed	No	Yes	English	European‐White	College or University (undergraduate)	None
P5: P	78	Male, Man	Retired	No	No‐India	English	South Asian	Graduate or professional degree	Heart disease, high blood pressure, cholesterol
P6: P and C	64	Female, Woman	Retired	No	Yes	English	European‐White	Some college or university	High blood pressure, Immune Thrombocytopenia
P7: C	42	Female, Woman	Employed	No	No‐India	English, Punjabi	Southeast Asian	Graduate or professional degree	None
P8: P	66	Male, Man	Retired	No	Yes	English	North American‐White	High school	Heart disease, cancer, arthritis
P9: P	73	Female, Woman	Retired	No	Yes	English	European‐White	Some college or university	High blood pressure, arthritis, chronic pain
P10: P	77	Female, Woman	Retired	Yes	Yes	English	North American‐White	College or University (undergraduate)	High blood pressure, heart disease, arthritis

### 3.2 Overview of Patient and Caregiver Work (Research Question 1)

Nine challenges were inductively identified from patient and caregiver narratives: (1) informational gaps from transferring information from one person to another, (2) confusion about content of information shared, (3) conflicting or confusing discharge instructions, (4) limited awareness about relevant community services and programmes, (5) feeling rushed and unprepared for discharge, with insufficient individualised training, (6) difficulty coordinating multiple appointments, services and equipment, (7) ambiguity about timing and follow‐up of community service, (8) uncertainty about abnormal symptoms and who to contact, and (9) new and existing community clinicians having incomplete or inaccurate information about patient information. Based on the challenges described, we interpreted them through the lenses of continuity of care and patient work.

Supplementary Table 1 outlines the types of work performed by patients and caregivers during hospital‐to‐home care transitions, which was used as an interpretive analytic lens. Participants described taking on various tasks across hospitalisation episodes. For those with a planned hospitalisation, before their hospital stay, they engaged primarily in logistical physical tasks and in cognitive tasks related to information collection and management. While in the hospital and preparing for discharge, participants engaged in physical tasks, including logistical tasks (e.g., purchasing home safety equipment), medication management (e.g., administering patient‐controlled analgesia as needed), and communication (e.g., waiting for updates). While in the hospital, some tasks required cognitive work, including logistical cognitive tasks (e.g., organising transportation), information collection and management, and information sharing. After leaving the hospital, the tasks requiring physical work included logistical, symptom and medication management, information sharing between patients and their caregivers and community‐based clinicians, and medical equipment management. Cognitive tasks involved emotional management, ongoing information collection about their health status and treatment plans, and management and financial arrangements. Based on participant descriptions, inadequate information, management, and relational continuity of care increased the burden on patients and caregivers during these transitions. The following sections address research question 2 regarding how continuity influenced the experience of work described above. The findings within each category were organised according to when the work occurred: before hospitalisation, during hospitalisation, and after discharge.

### 3.3 Theme 1: Physical and Cognitive Work Performed by Patients and Caregivers During Hospital‐To‐Home Transitions

Participants described tasks related to physical (subtheme 1a) and cognitive (subtheme 1b) work involved in managing their health during hospital‐to‐home transitions (see supplementary table 1). These sub‐themes are interpreted through a continuity of care lens to elicit insights into which forms of work present challenges address when seeking to improve transitions.

#### 3.3.1 Subtheme 1a: Physical Work During Hospital‐To‐Home Transitions

Participants described engaging in various types of physical work before, during and after hospitalisation. Participant 9, a patient who had a planned hospitalisation, explained that they engaged in logistical physical tasks, including preparing for discharge by purchasing necessary mobility and home safety equipment and arranging medications: ‘I went to my family doctor in advance and I said, look, I'm going to have this surgery…I asked her for a prescription, and so she gave me…hydromorphone.’ While a patient was in the hospital, patients' and caregivers' physical work included logistical tasks, such as managing personal items and belongings and coordinating transportation from the hospital‐to‐home in anticipation of discharge, and information sharing tasks, such as receiving written and verbal medical updates from clinicians. Participants indicated that the medical updates were often unplanned, leading them to wait hours for clinicians to provide updates. Physical work specific to caregivers included logistical tasks, such as visiting the patient while in the hospital: ‘Being there every day, day and night practically with him’ (Participant 6, a patient and caregiver). Some caregivers physically assisted them with self‐care tasks, which meant the caregiver had to clear/re‐arrange their own schedules to make time to do this (e.g., cancel or reschedule work or social events).

Participants engaged in more physical work after being discharged from the hospital than while in the hospital. Both patients and caregivers were engaged in logistical physical tasks. For instance, caregivers, if not physically present at the hospital, often had to drop everything to rush there when discharge occurred unexpectedly. Participants indicated they often received little notice of the discharge timing, as it depended on the completion of various hospital processes (e.g., medical examinations and administrative tasks). As a result, this made it harder for them to arrange tasks, such as logistical arrangements for transportation from the hospital‐to‐home.

You have to time the discharge and make sure you have the pills ready for the right time…the day that they [the patient] come home from the hospital is quite a scramble because…you have to go to the pharmacy…we've asked them to make sure…can we take home today's meds? Because the pharmacy might not have them ready till tonight.

(Participant 6, a patient and caregiver)

Some participants explained that they required physical assistance to help them get into the vehicle and to move around their homes due to their altered functional status. Those who lived alone had to seek alternative living arrangements, such as temporarily staying at a friend's house for support after discharge. Additionally, participants needed care from multiple clinicians in the community and from various settings, including in‐home visits and various clinics, which required transportation to attend. Attending clinic visits was challenging for some due to reduced mobility after hospitalisation. Participants explained that physically navigating their home and community environments after hospital discharge was emotionally and physically challenging: ‘because I…wasn't able to put any weight on my foot…with being non weight bearing, I didn't know how I would be able to get into my house and up the stairs to get into bed’ (Participant 9, a patient). Many participants reported having to wait for a home safety assessment after hospital discharge to make the necessary home safety modifications. Patients and caregivers expressed frustration with delays in receiving community services and were often left waiting for a phone call with limited information about when services would be provided, as these were typically scheduled after hospital discharge. Participant 6 (a patient and caregiver) expressed frustration with,

Waiting and not knowing when you were going to get a phone call [or] if you had to make that phone call to do the follow up or you're waiting around for that phone call to come in to set up an appointment to have someone come out…waiting around for the community care person or the care coordinator…to call and say, OK, we're sending a nurse out, but the nurse doesn't come out when they say that the nurse is coming out, and then when the nurse does come later, like we're not even talking the same day, they can't do anything because they don't have the proper equipment to deal with it.

#### 3.3.2 Subtheme 1b: Cognitive Work During Hospital‐To‐Home Transitions

Participants described engaging in various cognitive work before, during and after hospitalisation. Cognitive work before a planned hospitalisation involved information collection and management, such as arranging necessary care and contacting various clinicians for support. Participant 9, a patient, noted that some services, like a home safety assessment, were not available before discharge, prompting her to advocate for them in advance due to concerns about managing at home after discharge:

I was like trying to plan for my discharge in advance of being discharged…I phoned up the [home care service]…and I said I needed help in planning this and they said…you'll be fine and you'll just tell them at the hospital on the day of the operation. It's like if you need special specialised equipment, you can't just organise it after your operation.

Cognitive work during hospitalisation consisted of logistical tasks, such as organising transportation to and from appointments. Information collection and management tasks included staying updated about a patient's medical information. However, many participants reported challenges understanding medical jargon and acronyms. Patients also noted information sharing challenges when caregivers were not present during discussions with clinicians, and these conversations were rarely scheduled in advance. Some patients without caregivers indicated that medication side effects sometimes hindered their understanding of care instructions, resulting in difficulties with pain management and contributing to emotional frustration and physical discomfort (e.g., pain). Some participants explained that they used an online patient portal to receive medical updates, but not all information was available, and the portal did not allow them to ask questions, which left them to manage emotional tasks (e.g., stress or anxiety).

Another cognitive task in information collection and management involved determining who the right person was to answer questions about their care, since not all clinicians were authorised or equipped to provide updates. Nearly all participants took on emotional management tasks due to a lack of clarity about their discharge date and time, which complicated plans for transportation and medication pickup. Caregivers also faced emotional challenges related to the need to cancel important social events and manage the feelings of loved ones struggling to cope with hospitalisation. Some participants assumed the additional responsibility of seeking second opinions regarding the patient's diagnosis and medical status.

Every day was a bit of an anxiety to understand when you were going to get availability to the doctor to provide you the information you're requesting and very when the doctor did show up, you got the information, but it was more just the timeliness of it and the and the unpredictability of it was a constant.

(Participant 3, a patient)

Cognitive work after hospital discharge was considerable. The challenges encountered at this point led to emotional management tasks, as participants reported dealing with uncertainty and personal emotions. Participants had to manage logistics and collect and manage information from multiple clinicians and locations (e.g., at home and at various medical clinics). These tasks involved tracking the different appointments, their purposes, and the varying medical information provided by each clinician. One of the most challenging cognitive tasks reported by participants was updating community clinicians about their medical information since not all of them received this information from the hospital. Participants found this difficult because not all of them fully understood their medical information or could convey it in medical terminology, especially if it was not in writing. Other challenging cognitive tasks included determining whether their symptoms were normal or abnormal, and deciding whom to contact. While many patients received a contact number or were advised to visit the emergency room for abnormal symptoms, many did not understand what constituted ‘abnormal’ and were unsure if their experiences qualified as abnormal or were a typical part of recovery, particularly because patients were unfamiliar with what was considered normal for them.

Having him at home shortly after that, he had to obviously be monitored. He was put on blood thinners, and then he also takes it for seizures. So there was a balance of trying to manage his medications at that time with the changes that they've given.

(Participant 6, a patient and caregiver)

### 3.4 Theme 2: Patient and Caregiver Work Impacted by Continuity of Care

Theme 2 describes an interpretive mapping of participants' hospital‐to‐home experiences onto three types of continuity of care: informational, management and relational. Supplementary Table 2 illustrates significant gaps in continuity, which impacted patient and caregiver work during the hospital‐to‐home transition. Table 3 highlights key points of how continuity of care can be enhanced for older adults with complex care needs during hospital‐to‐home transitions.

**Table 3:** Supporting continuity of care for older adults with complex care needs and caregivers during hospital‐to‐home transitions.

Phase	How to support continuity of care during hospital‐to‐home transitions for older adults with complex care needs and caregivers
**Before hospitalisation (hospitalisation is planned e.g., elective surgery)**	‐Provide clear and tailored information on the transition process and care team/plan (e.g., equipment needs)
**In hospital before discharge**	**‐**Provide clear, consistent, tailored and timely information about the care plan and transition process (e.g., home safety and mobility, managing in the community)‐Clear and timely information to patients and caregivers about community services ‐Consistent care team and plan followed
**After hospital discharge**	‐24‐h emergency contact line provided with clear and timely instructions about use‐Patient and caregiver training on supporting care at home (e.g., medical equipment, follow‐up appointments, managing abnormal symptoms)‐Patient and caregiver receive clear information about available community services on portal and discharge summaries/booklets‐Follow‐up phone call after hospital discharge (ideally communication with a consistent care team member)‐Caregivers bridge gaps in relational continuity (e.g., share patient history and needs with new care team members in hospital and re‐instate relationship with community providers after hospital discharge)‐All care team members, including new and existing, are updated on patient details in a timely manner

#### 3.4.1 Subtheme 2a: Informational, Management, and Relational Continuity Impacted Physical and Cognitive Work for Patients and Caregivers Before Hospital Discharge

Based on the challenges described by participants, gaps in informational, management, and relational continuity appeared to increase their cognitive and physical work. Before discharge from the hospital, participants described informational gaps arising from information sharing about the patient's condition and care plan, which increased cognitive work. For instance, fragmented information sharing between patients and clinicians (i.e., informational continuity gap) resulted in physical tasks (e.g., patients and caregivers having to collect and manage additional information) and cognitive tasks (e.g., managing emotions such as anxiety due to the lack of a clear plan for managing the patient's care). For example, Participant 4 shared:

I would say the communication from the doctor wasn't always as readily available as we would have liked, you know. The doctor, of course, visits at different times. And especially when if a family member isn't able to be there and, and your loved one is hard of hearing, they don't remember everything…But so yeah, communication about the, the patient's current status.

(Participant 4, a caregiver)

However, when informational continuity was supported by patients and caregivers receiving relevant information (e.g., recommendations for home safety and mobility equipment, prompt updates on patient status, discharge information, and responses to their questions), participants reported a reduction in their cognitive workload. For example, as a caregiver, Participant 4 explained how being involved early reduced the cognitive work of discharge.

You're like if you can just tell us what you know what you're thinking of is the discharge date versus because then they'll say, OK, they're being discharged in 2 days…communication is really key…the more that the, the, the patients family can be involved, the better the helps to relieve the‐ the stress and tension…if the family member isn't able to‐ to actually chat with discharge coordinator, then it yeah, it just I would say then it creates some gaps in…full knowledge of the patient's needs when they come out.

(Participant 4, a caregiver)

There were disruptions in relational continuity during entry into the hospital and after discharge. As the care team changed, both patients and caregivers faced additional work, often needing to update and inform new care team members in the hospital and the community who were unfamiliar with the patient and their condition. Participant 8 shared an example that described gaps in relational continuity with a clinician who added cognitive work (i.e., emotional management due to lack of sustained relationship with surgeon):

We went to the pre‐admission meetings, and [the surgeon] explained…somewhat the procedure…My wife and I were not really clear. So we requested a meeting with the surgeon before, the before the surgery…we went into his office…for like an hour and he went through the whole procedure…So I had built it like a‐, a rapport with him…. Following the surgery with the complications, I never saw the surgeon, the actual surgeon, even once…I wish that. I could speak to the actual surgeon.

(Participant 8, a patient)

Another instance in which patients and caregivers reported that their physical and cognitive work decreased was when the hospital care team followed up with them after discharge or when their family doctor did. This communication provided an opportunity to seek further clarification and direction, reducing confusion and the need to locate additional sources of information.

#### 3.4.2 Subtheme 2b: Informational Continuity Impacted Physical and Cognitive Work for Patients and Caregivers After Hospital Discharge

Upon hospital discharge, gaps related to information were identified from participants' experiences. Patients' and caregivers' narratives exemplified a lack of informational continuity due to inadequate, conflicting, missing, or unclear information, and led to information overload. These informational and management continuity gaps tended to occur more when the information provided in the hospital was not tailored to their specific needs. As a result, patients and caregivers often felt confused about the physical care tasks they needed to manage at home, such as administering medication and monitoring for abnormal symptoms, as described by Participant 9:

Well, they [the clinicians] gave me lots of information…I ended up with 100 pages of printed material and it applied to all sorts of conditions other than mine. So it was a little confusing to know which parts I was supposed to be looking at. That would be particular to me, and‐ and then they‐ they set up an appointment for you at the Doctor like for maybe a‐ a week after. Yeah. And‐ and they [the clinicians] did give me a number. I think they gave me a number to call if‐ if I was worried or had any concerns, I guess if my foot was turning or something. But I think it was a pager number for the resident on call. OK. And‐ and, you never know exactly how much of an emergency you have to be in?

(Participant 9, a patient)

Another gap in informational continuity occurred when hospital information did not reach all community clinicians. This information discontinuity increased cognitive work for patients and caregivers. These information continuity issues created gaps in management continuity, leading to increased physical and cognitive work (e.g., unnecessary stress) for both patients and caregivers.

When informational continuity was supported, as exemplified by participants receiving clear, tailored, and timely instructions on crucial health‐related topics, such as signs of abnormal symptoms and whom to contact if issues arose after hospital discharge, their physical and cognitive work appeared to decrease. Participant 4 explained,

The first time my mother‐in‐law got out of the hospital, she was still quite…frail and she was on…. They [the clinicians] had recommended oxygen for her…We needed to rent a hospital bed…We, we went ahead and rented a hospital with bed and then she [clinician] told us the supervisor when she came to the house like…However, many days later, she [clinician] had said, oh, we could have covered that for you. Like so, but you know, so it was the information. There was a little bit of gap in like, oh, nobody at the hospital, people didn't tell us that.

(Participant 4, a caregiver)

#### 3.4.3 Subtheme 2c: Management Continuity Impacted Patient and Caregiver Work After Hospital Discharge

Gaps in management continuity following hospital discharge, such as ambiguity about the timing and follow‐up of community services and how care management tasks need to change as patients' conditions evolve over time, added physical and cognitive work for patients and caregivers, including additional information sharing tasks. Furthermore, gaps in management continuity increased the amount of cognitive tasks for patients, including emotional stress and the consumption of participants' time. For instance, Participant 6 expressed frustration with the uncertainty and delays related to community services after her knee replacement surgery. She described heightened anxiety from waiting for service arrangements added to the workload of patients.

Waiting and‐ and not knowing when you were going to get a phone call, if you had to make that phone call to do the follow‐up or if you're waiting around for that phone call to come in to set up an appointment to have someone come out…waiting around for the community care person to or the care coordinator, I guess to call and say, OK, we're sending a nurse out, but the nurse doesn't come out when they say that the nurse is coming out, and then when the nurse does come later, like we're not even talking the same day, they can't do anything because they don't have the proper equipment to deal with it. And mine was, I think, my knee replacement. I had an infection, so I needed to have nursing come in, check on the wound, you know, change everything and all that stuff. She didn't have the stuff to‐ to do the change with, so that delayed it even longer.

(Participant 6, a patient and caregiver)

Patients and caregivers explained that arranging community services while they were in the hospital and receiving details about their appointments would reduce their work after discharge. In addition, having a central point of contact to address their questions after hospital discharge would again reduce their cognitive and logistical workload. These exemplified a need to enhance management continuity to reduce patient and caregiver work.

## 4 Discussion

This qualitative study describes the physical and cognitive work that patients and caregivers perform during hospital‐to‐home care transitions and how informational, management, and relational continuity in care affect this work. As a study output, some study findings were translated into practical, actionable insights for system change through a vignette video to engage participants in the later co‐design workshops [47]. As shown in our analysis, the concepts of patient and caregiver work, and of continuity of care, overlap and intersect. If continuity is better supported, patients and caregivers may have to take on less physical and cognitive work. Our findings support prior literature highlighting the additional patient and caregiver work required when continuity of care is disrupted [19, 35]. The more gaps in continuity of care between hospital and community settings as well as within the same care setting (e.g., clinicians not all adhering to the same care plan), the more challenging it becomes for patients and caregivers to manage the transition. Furthermore, our findings align with previous research, which emphasises that the work involved in the transition from hospital‐to‐home for older adults with complex care needs is primarily a collective effort involving clinicians, patients and caregivers [26]. This suggests that patients without caregiver support may require additional help and attention to effectively manage after hospital discharge. For instance, relational continuity is often disrupted during and after hospitalisation because different clinicians provide care. Patients and caregivers often bridge these gaps in relational continuity by sharing patient history and needs with new care team members in the hospital and re‐establishing relationships with community clinicians after hospital discharge.

### 4.1 Adequate Training and Resources Are Needed to Support Patient and Caregiver Work During Transitions

Not all patient and caregiver work can be or should be avoided [22, 24]. There is an increasing emphasis on patients and caregivers being active partners in their healthcare, which requires them to perform health‐related work [26, 28, 48]. However, there is a lack of understanding about the actual work performed by patients and caregivers; this knowledge is necessary to inform the design of effective health systems [26]. Our study reveals that participants felt inadequately prepared to take on the additional tasks they were expected to as a result of gaps in the continuity of care, highlighting the need for reform in care transitions. This finding is transferable to care transitions in other healthcare settings, as models that examine patient‑ and caregiver‑focused outcomes (e.g., function, safety, and engagement) are increasingly recommended in addition to cost‑saving outcomes, which have traditionally been the focus of many hospital‑to‑home models [28, 49, 50]. When patients and caregivers lack the knowledge, resources, and skills needed, research shows this can lead to increased emotional burden and stress [25]. Some patients find health‐related work overwhelming, which can contribute to noncompliance with medical instructions and result in suboptimal health outcomes [25, 51, 52]. Therefore, it is important that patients and caregivers receive appropriate education and support, and have adequate skills, motivation, and confidence to actively participate in their care [28, 48]. As suggested in previous research, considering the ‘burden of treatment’ and patient work as indicators of quality of care may enhance care [22].

Task shifting is a strategy considered for its economic benefits, which involves ‘redistributing healthcare services’ to different care team members' [53]. Task shifting has been explored as a means to address workforce shortages and resource constraints [53] and may enhance care quality and continuity [54]. The specific strategies used in task shifting can vary by country and region, but training and collaboration are key components [53]. For instance, in Ontario, legislation allows regulated clinicians to delegate certain tasks to personal support workers, provided that the personal support workers receive training in performing the task for each patient, regardless of their prior experience with it [55]. A similar approach could be implemented for managing patient and caregiver work during hospital‐to‐home transitions. Future research could also explore how patients and caregivers perceive work during these transitions if they receive appropriate support, skills and resources.

Our study findings may be transferable to other healthcare settings. A review of 44 articles on transitional care, primarily from USA, Australia, Denmark, and China, highlighted the need to explore new methods for engaging and educating patients and caregivers [49]. Although caregivers often play a crucial role in supporting these transitions, their needs are frequently overlooked [49]. New approaches for evaluating patient and caregiver‐centred outcomes may be particularly important in universal healthcare contexts, where public funding can be a barrier and lead to a greater focus on reducing health system costs [56]. For example, the UK's discharge‐to‐assess model aims to shorten hospital stays by providing more community‐based supports; however, it can sometimes be challenging to effectively manage patient and caregiver expectations and needs, resulting in ‘wrong levels of care being provided’ and readmissions [56].

### 4.2 Digital Health Technology May Support Patient and Caregiver Work

Digital health technologies have been proposed as a solution to improve care continuity and reduce the work of patients, caregivers and clinicians [57, 58]. Specific digital health solutions have been proposed to support older adults with complex care needs during hospital‐to‐home care transitions [59]. However, if these interventions are inefficient, they risk inadvertently increasing workload and negatively affecting patient and clinicians' outcomes [60, 61]. Thus, the information generated by this study can inform the development of digital health solutions that support continuity while reducing patient and caregiver workload. Future research is needed to explore how to leverage digital technologies to bridge relational continuity, as relational continuity is least supported by digital health interventions supporting hospital‐to‐home transitions [4].

Our study has several limitations. The sample predominantly consisted of individuals with post‐secondary education, higher household income, and English fluency, which limits the transferability of findings to populations facing socioeconomic disadvantage, lower health literacy, or language barriers. This is a significant limitation because research has shown that both person‐ and context‐related factors, such as education and language, affect patient outcomes during care transitions [62, 63, 64, 65]. Furthermore, individuals facing challenges with medical affordability or language barriers may experience even greater workloads and vulnerability during hospital‐to‐home transitions [26]. Additionally, data were collected during the COVID‐19 pandemic. While not all participants experienced their transition during this time (some had already done so before the pandemic), the restrictions may have created additional challenges for some, potentially intensifying patient and caregiver work. We did not impose a time restriction on participants' hospital‐to‐home transition experiences to enhance recruitment feasibility, as it can be challenging to recruit individuals during this difficult period [66]. However, we recognise this decision may have led to our findings reflecting varied participant experiences, as these experiences can differ with changes in the healthcare system, policies, and service provisions over time (e.g., before or during the COVID‐19 pandemic). Finally, analytic decisions, such as grouping emotional and cognitive work, may have underemphasised the emotional burden during care transitions. These factors should be considered when interpreting the findings.

## 5 Conclusion

In conclusion, our study findings highlight the physical and cognitive work undertaken by both patients and caregivers during the transition from hospital‐to‐home, particularly when care continuity is disrupted. Our findings suggest that improving informational, management, and relational aspects of care continuity could reduce patient and caregiver burden. Digital health technologies, individualised training and resources for patients and caregivers and joint visits between hospital and community clinicians may improve continuity. Additionally, there is a need for transitional care interventions to better prepare older adults with complex care needs and their caregivers for the challenges they experience during transitions.

## Author Contributions

**Hardeep Singh:** writing – original draft, data curation, formal analysis, writing – review and editing, methodology. **Michelle L. A. Nelson:** funding acquisition, conceptualisation, supervision, resources, writing – review and editing, methodology. **Terence Tang:** writing – review and editing, supervision, funding acquisition, conceptualisation, methodology. **Kerry Kuluski:** writing – review and editing, funding acquisition, methodology. **Anie Udofia:** validation, formal analysis. **Hedieh Molla Ghanbari:** writing – review and editing, conceptualisation, funding acquisition. **Ingryd Cunha Ventura Felipe:** project administration, writing – review and editing. **Jason Nie:** writing – review and editing, methodology, funding acquisition. **Kednapa Thavorn:** conceptualisation, writing – review and editing, methodology, funding acquisition. **Robert J. Reid:** writing – review and editing, methodology, funding acquisition. **Walter Wodchis:** methodology, writing – review and editing, funding acquisition. **Carolyn Steele Gray:** conceptualisation, funding acquisition, investigation, methodology, validation, writing – review and editing, formal analysis, supervision.

## Ethics Statement

Research ethics approval for this study was obtained from Sinai Health Systems.

## Consent

All participants provided informed verbal consent prior to data collection.

## Conflicts of Interest

The authors declare no conflicts of interest.

## Supporting information

Supporting File

## Data Availability

The data that support the findings of this study are available on request from the corresponding author. The data are not publicly available due to privacy or ethical restrictions.
