# Supplementary material for: Exploring Patient and Caregiver Work and Continuity of Care During Hospital‐to‐Home Transitions of Care in the Canadian Context
Source: Health Expect. 2026 Jun 18;29(3):e70727. doi: 10.1111/hex.70727 (PMC13280220; doi:10.1111/hex.70727)
Supplement: Supplementary file 1 — Supporting File [file HEX-29-e70727-s001.docx]

| **Before hospitalization (situations where hospitalization is planned, such as an elective surgery)** | |
| --- | --- |
| **PHYSICAL WORK** | **COGNITIVE WORK** |
| ***Logistical physical tasks:*** preparing for post-discharge by purchasing recommended adaptive equipment such as a toilet seat, bath seat, knee scooter (patient and caregiver) | ***Information collection and management tasks***: Figuring out who to seek support from to make preparatory arrangements (e.g., family doctor for medication changes, occupational therapist for home safety assessment) |
| **In the hospital before discharge** | |
| **PHYSICAL WORK** | **COGNITIVE WORK** |
| ***Logistical physical tasks***: figuring out how to transfer in and out of the vehicle post-surgery with limited mobility, with physical assistance from a friend, preparing for post-discharge by purchasing recommended adaptive equipment such as a toilet seat, bath seat, knee scooter, visiting the patient in the hospital, being present in the hospital on the day of discharge to physically support discharge  ***Medication management tasks***: self-administering patient-controlled analgesia as needed, based on pain levels and dosing plan  ***Communication tasks***: waiting for a phone call in case the patient needed communication support while in the hospital | ***Logistical cognitive tasks***: organizing transportation, rescheduling or declining social engagements e.g., wedding invitation, preparing for discharge, planning for physical support post-discharge and organizing transportation  ***Information collection and management tasks***: staying updated about patients’ health status and progress, exploring alternative treatments in different countries or seeking a second opinion, reviewing information provided in the hospital about what to expect upon returning home, monitoring and interpreting medical updates, determining transportation home from the hospital  ***Information sharing tasks***: helping the patient communicate with clinicians to receive health and treatment updates and seek clarity on care plans, determining which questions to ask clinicians, sharing the diagnosis and updates with other family members and friends, and advocating for home care services  ***Emotional tasks***: managing uncertainty, coping with diagnosis, supporting other loved ones struggling to cope, feeling unprepared for discharge due to uncertainty and anxiety about managing physical needs at home |
| **After hospital discharge** | |
| **PHYSICAL WORK** | **COGNITIVE WORK** |
| ***Logistical physical tasks***: visiting a friend’s house for physical support post-discharge, arranging transportation and attending to and from appointments in the community, receiving homecare services (being present and available), arranging mobility/home safety equipment by researching vendors, delivery dates, costs, rental options, and availability for equipment pick up if delivery is not available, arranging post-discharge medication  ***Symptom and medication management physical tasks***: Seeking help for symptoms that are abnormal (e.g., contacting emergency services), managing pain control, tracking/monitoring the patient’s status, administering updated medication regimens, managing symptoms of concurrent illnesses of other family members  ***Information sharing physical tasks***: receiving a check-in phone call after hospital discharge, updating and communicating with various community clinicians about medical information and hospital visits  ***Mobility and home safety physical tasks:*** navigating post-hospital mobility: learning to use a new mobility aid, modifying home environment for safety and figuring out how to enter and navigate the home with changes in mobility  ***Medical equipment management physical tasks***: supporting the patient after discharge physically (e.g., oxygen tank) | ***Emotional tasks***: managing uncertainty about follow-up services related to when and if they will call or if the patient/CG should initiate contact (e.g., 2-3 weeks or 6-10 weeks post-discharge), dealing with personal emotions (frustration, emotional upset, worry) related to the patient’s status post-discharge and service quality, coping with feelings of medical negligence  ***Logistical tasks***: Tracking different appointments and arranging transportation  ***Information collection and management of cognitive tasks***: determining who to call if abnormal symptoms occur, reviewing medical information through the patient portal and asking follow-up questions, figuring out how to follow the medication regimen, reviewing extensive post-discharge information, navigating gaps in medical information, finding and determining eligibility for community services, recalling verbal discharge instructions, determining and arranging home care services, understanding medication regimens  ***Symptom management:*** determining whether their symptoms were normal or abnormal and deciding whom to contact.  ***Financial arrangements****:* making financial arrangements for rehabilitation services  ***Information sharing tasks:*** advocating for the patient if home care staff are disrespectful |

Supplementary Table 1: Patient and caregiver work during hospital to home transitions

| **Gaps in continuity of care** | **Patient and caregiver work** |
| --- | --- |
| **Before hospitalization (situations where hospitalization is planned e.g., elective surgery)** | |
| ***Informational continuity gap***: information about required equipment not available prior to hospitalization | **PHYSICAL WORK**  ***Logistical physical tasks:*** purchase recommended adaptive equipment (e.g., toilet seat, bath seat, knee scooter) |
| ***Informational and management continuity gap***: lack of information regarding transition process and their care team and care plan | **COGNITIVE WORK**  ***Information collection and management tasks***: figure out sources of support for preparatory tasks (e.g., family doctor for medication changes, occupational therapist for home safety assessment) |
| **In hospital before discharge** |  |
| ***Informational continuity gaps***: insufficient information sharing (e.g., car transfers, pain medication dosing, patient progress)  ***Management continuity*** ***gaps***: inconsistent care plan; limited logistical support (e.g., arranging transportation home from hospital); unclear referral and follow-up for homecare services | **PHYSICAL WORK**  ***Logistical physical tasks***: learn vehicle transfer techniques (patients) and provide physical support on discharge day (caregivers); purchase recommended adaptive equipment (e.g., toilet seat, bath seat, knee scooter)  ***Medication management tasks***: manage pain medication based on pain levels and dosing  ***Information sharing tasks***: information sharing with hospital staff |
| ***Informational continuity gaps***: lack of timely communication with clinicians about patient’s status; some information continuity gaps caused by complex medical terminology, medication side-effects impacting patient memory, accessibility (hearing), information overload, or clinician uncertainty (e.g., pending medical investigations)  ***Informational and management*** ***continuity*** ***gaps***: test results and next steps not clearly communicated to patients and caregivers; managing conflicting information received from different clinicians regarding the patient’s status and symptoms  ***Management and relational continuity gaps***: some clinicians (often who were new patients to care team) in the hospital and community were unfamiliar with the patient's condition or status  ***Relational continuity gaps***: lack of consistent relationships over time with clinicians, with some clinicians seen only once (e.g., surgeon); multiple staff change during care without consistent care team | **COGNITIVE WORK**  ***Logistical cognitive tasks***: modify personal schedule; prepare for discharge (e.g., arrange transportation or community support)  ***Information collection and management tasks***: stay updated about patient’s health status (collect, understand and review information from different sources), explore treatment options, plan discharge logistics, support information sharing with new and existing care team and advocating for patient needs  ***Emotional tasks***: manage own emotions (e.g., uncertainty and anxiety about managing at home, coping with diagnosis); provide emotional support to loved ones |
| **After hospital discharge** | |
| ***Informational continuity gaps***: information not tailored to patients causing difficulty applying information about abnormal symptoms to personal symptoms (e.g., abnormal pain levels); limited information on post-discharge mobility; uncertainty regarding community service initiation (e.g., follow-up appointment timings), managing medical equipment at home and available funding for eligible services and equipment  ***Management continuity gaps***: lack of timely sharing of medical plan/summary from hospital to community care team  ***Management continuity gaps***: unclear or inconsistent medication plan caused confusion; patient and caregiver preferences not integrated into care plans | **PHYSICAL WORK**  ***Logistical physical tasks***: arrange physical support post-discharge, transportation to and from community service appointments; receive homecare services (be present and available), arrange mobility/home safety equipment (e.g., finding vendors, coordinating delivery dates, costs, rental options, and equipment pick up); arrange post-discharge medication  ***Symptom and medication management physical tasks***: monitor symptoms and status and seek support; medication and symptom management; manage concurrent caregiving tasks  ***Healthcare communication physical tasks***: receive a follow-up call after hospital discharge and information sharing with community clinicians  ***Relational gaps***: caregivers had to re-establish relationships with community clinicians (e.g., family doctor) and update them about hospital-based care  ***Mobility and home safety physical tasks:*** navigate post-hospital mobility (e.g., using new mobility aid, modifying home environment)  ***Medical equipment management physical tasks***: supporting the patient after discharge physically (e.g., oxygen tank, self-care) |
| ***Informational continuity gaps***: uncertainty regarding community care follow-up; limited information on financial and community support; lack of understanding about medication instructions due to medical jargon, information not personalized; lack of timely information about patient updates; unhelpful 24-hour emergency line directed patient elsewhere  ***Management continuity gaps***: lack of unclear which clinicians to follow-up with | **COGNITIVE WORK**  ***Emotional tasks***: managing uncertainty about follow-up services, handling personal emotions (e.g., frustration, emotional upset, worry) related to patient’s status and service quality, coping with feelings of medical negligence  ***Logistical tasks***: tracking appointments and arranging transportation  ***Information collection and management cognitive tasks***: identify who to contact for abnormal symptoms, review medical information, understand medication regime, review post-discharge information, navigate gaps in medical information, find and determine eligibility for community services, recalling verbal discharge instructions, coordinate homecare services  ***Symptom management:*** assess if symptoms are abnormal and decide whom to contact  ***Financial arrangements****:* organize finances for equipment and services not covered  ***Communication tasks:*** advocate for the patient if care team not meeting patient needs |

Supplementary table 2: Patient and caregiver work aligned to gaps and support of continuity of care
